# Resistance training increases myofibrillar protein synthesis in middle-to-older aged adults consuming a typical diet with no influence of protein source: a randomized controlled trial

**DOI:** 10.1016/j.ajcnut.2025.04.019

**Published:** 2025-04-25

**Authors:** Marie Korzepa, Jonathan I Quinlan, Ryan N Marshall, Lucy M Rogers, Archie E Belfield, Yasir S Elhassan, Alex Lawson, Chloe Ayre, Joan M Senden, Joy PB Goessens, Elisa I Glover, Gareth A Wallis, Luc JC van Loon, Leigh Breen

**Affiliations:** 1 School of Sport, Exercise and Rehabilitation Sciences, University of Birmingham, Birmingham, United Kingdom; 2 NIHR Birmingham Biomedical Research Centre, University Hospitals Birmingham NHS Foundation Trust, United Kingdom; 3 Intsitute of Metabolism and Systems Research, University of Birmingham, Birmingham, United Kingdom; 4 Biochemistry Department, Heartlands Hospital, Birmingham, United Kingdom; 5 Department of Human Biology, NUTRIM, Maastricht University Medical Centre+, Maastricht, The Netherlands; 6 Specialised Nutrition Department, Volac Whey Nutrition Limited (Arla Food Ingredients Group), Royston, Hertfordshire, United Kingdom

**Keywords:** animal protein, plant protein, resistance exercise, muscle anabolism, sarcopenia, human physiology

## Abstract

**Background:**

The primary protein source of a diet may impact skeletal muscle maintenance with advancing age. The impact of the animal and plant protein contents of a typical protein-containing diet on muscle anabolism in middle-to-older aged adults is unknown.

**Objectives:**

To determine muscle adaptive remodeling response to a 10-d dietary intervention containing divergent protein sources, with and without resistance exercise training (RET) in middle-to-older aged adults.

**Methods:**

In a single-blind randomized controlled trial, 27 50- to 70-y-old participants consumed 1.0 g·kg BM^−1^·d^−1^ of protein from an animal-focused whey protein–supplemented diet (AW-D) or plant-focused pea protein–supplemented diet (PP-D). Throughout the 10-d diet intervention, unilateral knee extensor RET was performed every other day. Deuterated water ingestion and skeletal muscle biopsies enabled measurement of daily integrated myofibrillar protein synthesis (iMyoPS) rates in the trained and untrained legs. Changes in metabolic rate, body composition, lipid profiles, renal function, whole-body nitrogen balance (WBNB), strength, and muscle architecture were also determined.

**Results:**

Daily iMyoPS rates were significantly greater (*P* < 0.001) in the trained leg compared with the untrained leg for AW-D (1.44 ± 0.26 vs. 1.29 ± 0.27 %⋅d^−1^) and PP-D (1.50 ± 0.17 vs. 1.34 ± 0.21 %⋅d^−1^) with no differences between groups, within leg. Training and diet did not affect intracellular anabolic signaling, muscle architecture, strength, metabolic rate, renal function, or WBNB. Serum non–HDL-cholesterol was significantly (*P* = 0.014) lower following the intervention for PP-D only (pre: 3.89 ± 0.84; post: 3.37 ± 0.78 mmol⋅L) with no other changes in lipid profiles.

**Conclusions:**

The 10-d provision of 1.0g·kg BM^−1^·d^−1^ from predominantly plant-derived or animal-derived protein does not influence daily iMyoPS rates in middle-to-older aged adults and has little impact on metabolic and renal health parameters. RET enhances rates of daily iMyoPS in middle-to-older aged adults consuming a typical protein-containing diet, with no influence of protein source.

**Clinical Trial Registry number:**

ClinicalTrials.gov NCT05574205 (https://clinicaltrials.gov/study/NCT05574205).

## Introduction

The loss of skeletal muscle mass and function with aging (sarcopenia) reduces quality of life and increases risk of falls, comorbidities, and mortality [1,2]. Sarcopenia is thought to commence from the fourth decade of life [3], although most research studies have focused on those aged >60 y. Resistance exercise training (RET) and adequate dietary protein nutrition are well-characterized muscle anabolic stimuli to support muscle health with advancing age [4,5]. However, compared with younger adults, the muscle protein synthesis (MPS) response to these anabolic stimuli is blunted in older adults [[6], [7], [8]] and may underpin sarcopenia progression. Evidence suggests that the dysregulation of MPS may manifest in middle age [9,10].

To overcome age-related muscle anabolic resistance, it is suggested that older adults require higher per-meal protein intakes compared with younger adults [[11], [12], [13]]. In agreement with these recommendations, higher total daily and per-meal protein intakes are associated with greater muscle mass retention in older adults [14,15]. However, consuming a higher-protein diet in older age is challenging due to a lack of awareness of protein requirements [16], the satiating properties of protein-dense foods [17], and impairments in appetite [18] and olfactory/gustatory functions [19,20].

Considering the challenges associated with achieving high-protein diets, focus has shifted to the importance of protein source to support muscle anabolism and maintenance in older age. The quality of a protein source is largely based on the capacity to support whole-body amino acid requirements [21]. The importance of protein source/quality for muscle anabolism centers on the bioavailability of essential amino acids (EAAs); determined by the constituent EAA profile and digestibility of the protein source [22]. Animal-derived proteins have been considered to be of higher quality than most plant-derived proteins in their capacity for MPS stimulation, due to greater EAA abundance and digestibility [23]. The superiority of animal- over plant-derived protein intake for MPS stimulation in older adults has been demonstrated with supplements [24] and a whole-food mixed meal [25], albeit under acute experimental conditions. Over long-term free-living conditions, equivalent rates of integrated myofibrillar protein synthesis (iMyoPS) and mixed MPS have been shown in healthy older adults consuming high-to-moderate protein-containing vegan and omnivorous diets [26,27]. However, the importance of dietary protein source for muscle metabolic regulation in older adults may be more apparent in typical protein-containing diets [28,29]. Furthermore, the addition of protein supplements to suboptimal protein-containing meals may be a feasible way to increase protein intake and postprandial MPS stimulation [30]. At present, it is unclear whether altering the source of ingested protein through supplementation in the typical omnivorous diet of middle-to-older aged adults impacts rates of daily iMyoPS at rest and with RET.

The present study determined the impact of a 10-d omnivorous diet providing 1.0 g·kg BM^−1^⋅d^−1^ of primarily animal- or plant-derived protein through supplements and whole foods, alone and in combination with RET, on daily iMyoPS rates in middle-to-older aged adults. Exploratory secondary outcomes included measures of body composition, muscle morphology, strength, and markers of metabolic and renal health. We hypothesized that an omnivorous diet providing a higher proportion of animal-derived protein would support greater rates of daily iMyoPS rates in middle-to-older aged individuals compared with a higher plant-derived protein-containing diet, in both rested and trained legs, with no adverse/differential effects on secondary outcomes between conditions.

## Methods

### Participants and ethical approval

Twenty-seven middle-to-older aged volunteers participated and completed the study. Initial recruitment was conducted through local advertisements, and participants were screened for eligibility in person after initial contact via e-mail or telephone. Exclusion criteria included *1*) aged <50 or >70 y, *2*) recent engagement in structured exercise training, *3*) metabolic conditions, respiratory disease, or chronic illness, *4*) habitual smoking, *5*) allergies or intolerances to study materials and supplements, *6*) use of medications known to affect muscle protein metabolism, and *7*) following an exclusively animal- or plant-based diet. Ethical approval was acquired from Surrey Boarders Research Ethics Committee (21/LO/0401). This trial was registered at ClinicalTrials.gov (NCT05574205). All procedures were carried out in line with the Declaration of Helsinki (7th edition). Participants were informed of the study purpose, experimental procedures, and potential risks associated with participating before they provided written informed consent. All visits were carried out at the University of Birmingham, School of Sport, Exercise and Rehabilitation Sciences, United Kingdom. Sample size estimates were based on previous work from others [31] measuring 3-d iMyoPS with unilateral exercise and comparing diets supplemented with additional leucine (emulating higher quality protein) versus no leucine supplementation (lower quality protein) with *n* = 10 for each group. We estimated using G^∗^Power (version 3.1) that to detect a difference in iMyoPS between high- and mixed-quality protein diets and exercised and nonexercised legs in older adults (alpha = 0.05; beta = 0.95), a sample size of 12 per group provides power to detect the smallest critical value of change in iMyoPS of 5% to 15% between legs and/or dietary interventions.

### Study design

**FIGURE 1:**
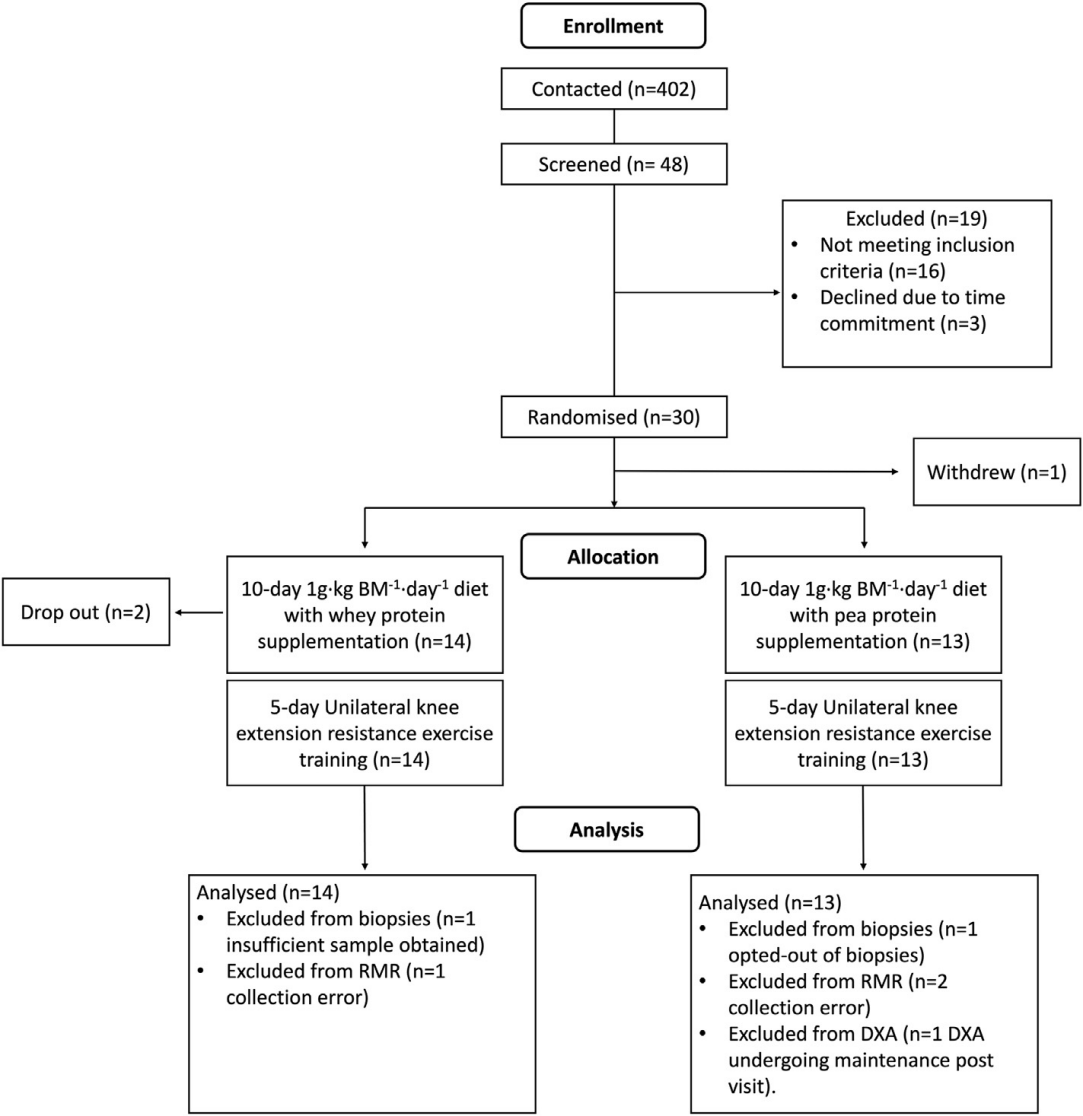
CONSORT flow diagram. BM, body mass; DXA, dual-energy X-ray absorptiometry; RMR, resting metabolic rate.

**FIGURE 2:**
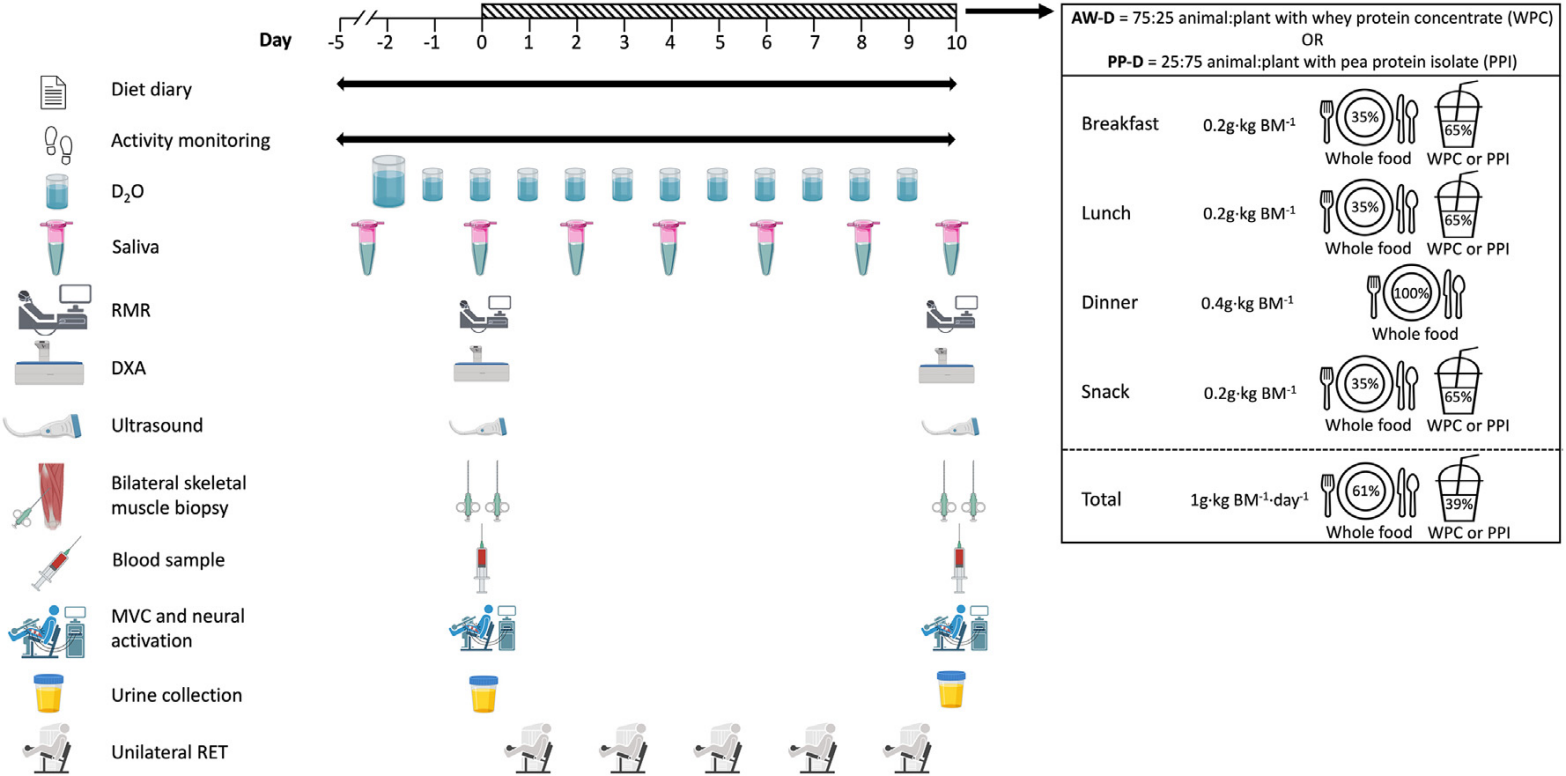
Schematic overview of the study timeline. Overview of the study design with 5-d habitual measurement phase and 10-d intervention phase of controlled diet and RET. BM, body mass; AW-D, animal-focused whey protein–supplemented diet; D_2_O, deuterated water; DXA, dual-energy X-ray absorptiometry; MVC, maximal voluntary contraction; PP-D, plant-focused pea protein–supplemented diet; RET, resistance exercise training; RMR, resting metabolic rate.

Upon study induction, participants were randomized, according to a predefined sex-stratified participant code, to a 10-d controlled dietary intervention providing ∼1 g·kg BM^−1^·d^−1^ of protein with an animal-focused whey protein–supplemented diet (AW-D; *n* = 14) or plant-focused pea protein–supplemented diet (PP-D; *n* = 13). Due to the complexity and specificity of the dietary protein provision in AW-D and PP-D, the trial operated as single-blind randomized controlled trial. Throughout the intervention, supervised unilateral leg extension RET was undertaken every other day. Based on previous comparisons of the iMyoPS response to shorter-term dietary supplementation and/or RET interventions [32,33], 10 d were deemed sufficient to detect potential differences in the rate of daily iMyoPS between AW-D and PP-D. A 10-d time frame also provides a closer representation of true free-living conditions in middle-to-older aged adults compared with previous studies [31,34,35]. See the CONSORT diagram in Figure 1 for participant flow through the protocol and Figure 2 for a schematic overview of the study.

### Habitual characteristics (day −5)

Following study induction, participants were provided with a wrist-worn accelerometer (GENEActiv; ActivInsights) set to record activity at 60 Hz over 15-d to determine physical activity prior to and throughout the 10-d intervention. Participants were also instructed to fill out a detailed weighed food diary for a minimum of 3-d (including the day before the intervention and 1 weekend day) to evaluate habitual dietary intake. Anthropometrics were measured for body mass (OHAUS) and height (SECA), whereas body fat percentage was determined using bioelectrical impedance analysis (BIA; BC-420MA; TANITA). Preliminary anthropometrics were used to calculate dietary protein intake at 1.0 g⋅kg BM^−1^·d^−1^ for the subsequent 10-d intervention phase and estimated daily energy requirements according to the Harris–Benedict equation [36] and multiplied by a physical activity correction factor rated for each participant.

### Preliminary assessments (day −2)

#### Knee extensor strength

After 3 d of dietary intake and physical activity monitoring, participants reported to the laboratory for an assessment of unilateral maximal knee extensor strength on a knee extension machine (Matrix Fitness). For all participants, the leg to be trained during the 10-d diet intervention was randomized and counterbalanced between AW-D and PP-D groups according to leg dominance. Participants were instructed on the correct technique to encourage a full range of motion to promote full concentric to eccentric contraction of the knee extensors. Participants initially carried out a warm-up set of 10 repetitions at a low load (14–29 kg). The estimation of 1 repetition maximum (1RM) was determined by progressively increasing the load by 7–9 kg and asking the participant to complete 3 full extensions with 2 min between each weight increase. The estimated 1RM was determined when the load where full extension was not achieved or 3 repetitions could not be completed [37]. The machine set-up for each participant was documented for replication in later training visits.

#### Deuterium oxide tracer protocol

Deuterium oxide (D_2_O) tracer (70% ^2^H_2_O; Cambridge Isotope Laboratories) was consumed daily over a 12-d period to achieve ^2^H enrichment of the body water pool at ∼0.7 atom percent excess (APE; [38]) for the calculation of free-living daily iMyoPS rates (described later). The use of D_2_O has been validated as a measure of free-living daily iMyoPS and for detecting between-leg differences with unilateral RET [38,39] and dietary supplementation interventions [35,40]. Following 1RM strength assessment, participants provided a baseline (i.e., unenriched) saliva sample and then consumed a 400 mL D_2_O loading dose provided in 8 × 50 mL boluses, with the first 3 doses given ≥60 min apart to mitigate potential side effects (i.e., nausea, dizziness, blurred vision), which can occur with sudden and drastic incorporation of D_2_O into the body water pool. Thereafter, participants were instructed to consume the remaining 5 × 50 mL D_2_O doses at home, spaced 60 min apart. Participants were asked to document the time D_2_O consumption was completed and also document any side effects. For the next 11 d (ending on day 10 of the intervention), participants provided a daily saliva sample (∼10 mL) upon waking before consuming a 50 mL top-up dose of D_2_O (until day 9). A slight upward drift in ^2^H enrichment was expected given the absolute D_2_O dose that was provided, as previously observed [38]. D_2_O consumption was well tolerated with no side effects reported beyond minor transient dizziness.

### Preintervention and postintervention measures (days 0 and 10)

#### Resting metabolic rate

Two days following 1RM strength assessment, participants reported to the laboratory after a >10 h overnight fast and abstaining from strenuous exercise for the prior 24 h to complete a series of measures and begin the 10-d diet intervention. All measures described later were repeated the day immediately following the 10-d diet intervention. Participants first had resting energy expenditure and respiratory exchange ratio (RER) measured by laying in a supine position while continuously wearing a Hans–Rudolf face mask (Cranlea) over a 20-min period (following acclimation) to collect breath-by-breath measurements through a Vyntus Jaeger CPX metabolic cart (Vyarie Medical), as previously described [41].

#### Muscle architecture

Ultrasonography of both legs was undertaken to determine structural alterations of the m.*Vastus Lateralis*. Laying supine, femur length was measured by locating the lateral epicondyle of the knee and protrusion from the greater trochanter, and the midpoint was marked. The ultrasound probe (LOGIQ; M-18, 5 cm) was moved around this midpoint to find the borders of the m.*Vastus Lateralis.* Once the borders were located, the probe was placed parallel to the leg and manipulated, and depth was adjusted to ensure clear aponeurosis and fascicles of the m.*Vastus Lateralis* were visible. A series of images were captured on LOGIQ S8 (General Electric) ultrasound at a scan depth of 4–5 cm and frequency of 3–13 MHz and saved for each leg when there was minimal pressure from the probe onto the leg. Images with clear aponeurosis and fascicles were exported for analysis of muscle thickness and pennation angle. All representative images were analyzed on semiautomated analysis on Fiji ImageJ (V1.54; US National Institutes of Health) using Simple Muscle Architecture plugin (version 2.21) [42].

#### Dual X-ray absorptiometry scanning

Dual X-ray absorptiometry (DXA) scanning (Discovery A; Hologic) was used to determine whole -body and regional fat mass and fat-free mass (FFM). Participants were laid in the supine position with feet taped to point toward the midsagittal plane to maximize the length of the femur head on both legs. Prior to DXA-derived body composition analysis, body mass and total body water were measured through BIA, using a 4-compartment model to account for any body mass difference due to total body water preintervention and postintervention.

#### Skeletal muscle biopsies

Percutaneous skeletal muscle biopsies were taken from the *m.Vastus Lateralis* of both legs to allow for within and between-leg comparisons in daily iMyoPS rates with 10-d diet interventions under conditions of rest and exercise. Skeletal muscle biopsy procedures were performed under local anesthetic (1% lidocaine hydrochloride) using the Bergström technique adapted for suction [43]. The yield of skeletal muscle tissue for iMyoPS fractional synthesis rate (FSR; ∼50–80 mg) was freed of visible connective tissue and fat, placed into a cryovial, and immediately snap-frozen in liquid nitrogen. Snap-frozen tissue was stored at −80°C until later analysis (detailed later).

#### Neural activation and dynamometry

The strength of both legs was assessed by isometric maximal voluntary contraction (MVC) using Biodex dynamometer (Biodex Medical Systems). Participants were seated at 85˚ with chair height adjusted until the lateral epicondyle of the knee was in line with the pivot point of the leg attachment. The positioning of the Biodex was recorded for each participant and replicated for postintervention measures. To carry out the interpolated twitch, disposable electrodes were placed on and adjacent to the superficial femoral nerve and the voltage set to evoke a small involuntary contraction upon short-term stimulation. Participants carried out a short warm-up followed by 3 short MVCs each >45 s apart to allow for phosphocreatine resynthesis and minimize acute fatigue. During and immediately after each MVC, an electrical impulse was delivered to ascertain the superimposed and potentiated twitch to find voluntary activation (VA), as previously described [44]. All dynamometry assessments were performed following muscle biopsy procedures so as not to interfere with acute intramuscular signaling events.

#### 24 h Urine collection

Participants were provided with 2 urine containers (3 L capacity) to collect all passes over days 0-1 and days 9-10 of the intervention. Urine collection at these time points was used to estimate whole-body nitrogen balance (WBNB) change during the 10-d diet interventions. The time between the first and last pass of urine collection over the 24 h period was recorded by the participant, and the total urine volume was measured in the laboratory. Two 15 mL urine samples were aliquoted, treated with 2 M sodium azide (75 μL per 2 mL of sample), and centrifuged at 4°C at 4000 *g* for 20 min. From this, aliquots of 2 mL were frozen at −80°C to minimize bacterial growth ready for later analysis of urea and creatinine to calculate WBNB (described later).

### Dietary intervention

AW-D and PP-D were designed to provide a total of ∼1.0 g·kg BM^−1^·d^−1^ of protein in line with the typical dietary protein intakes of middle-to-older aged adults [29,45], which are beyond the recommended dietary allowance of 0.8 g·kg BM^−1^·d^−1^ but below alternative requirements to support skeletal muscle health of these individuals [46]. The distribution of dietary protein was 0.2 g·kg BM^−1^ at breakfast, lunch, and midafternoon snack, and 0.4 g·kg BM^−1^ at dinner. The total daily protein intake and distribution pattern were designed to mimic the typical eating habits of older adults [29]. At breakfast, lunch, and midafternoon snack, ∼0.13 g·kg BM^−1^ (65%) of the ingested protein was provided in the form of a smoothie containing supplemental whey protein concentrate (WPC; Volactive UltraWhey Sugar Free; Volac) for AW-D or pea protein isolate (PPI; MyProtein; The Hut Group) for PP-D. Both supplemental sources are relatively of high quality considering the refined protein content and higher digestibility due to their powdered form. However, in the context of the 10-d dietary intervention, PPI increased the proportion of plant provision and contained fewer EAAs compared with the animal-derived WPC (Table 1). Participants were shown typical food items, which would be consumed as part of the 10-d dietary intervention and were allowed to select their food preferences. Some flexibility in elements of the dietary control was permitted to improve adherence and the inclusion of representative and diverse dietary preferences. The specific timing of meal/snack consumption was at the discretion of the participants to minimize attrition. Timings of food consumption were documented by participants in a written dietary log, where participants could also disclose if any additional foods were consumed or if any of the provided foods remained leftover. With the exception of alcohol, drinks were not restricted during the diet intervention. To control for protein, participants were provided with a measured amount of milk to add to caffeinated/warm beverages and factored into daily protein provision.

**TABLE 1:** Relative daily amino acid provision from supplemental sources.

Daily intake^1^ (g)	Whey protein concentrate (Volactive sugar free)	Pea protein isolate (MyProtein)	Between groups (*P* value)
Aspartic acid	2.52 ± 0.35	2.61 ± 0.60	0.653
Serine	1.24 ± 0.17	1.21 ± 0.28	0.753
Glutamic acid	4.0 ± 0.56	3.63 ± 0.83	0.182
Glycine	0.46 ± 0.06	0.91 ± 0.21	<0.001^2^
Histidine	0.40 ± 0.06	0.52 ± 0.12	0.003^2^
Arginine	0.55 ± 0.08	1.78 ± 0.41	<0.001^2^
Threonine	1.57 ± 0.22	0.78 ± 0.18	<0.001^2^
Alanine	1.16 ± 0.16	0.92 ± 0.21	0.003^2^
Proline	1.38 ± 0.19	1.01 ± 0.23	<0.001^2^
Cysteine	0.55 ± 0.08	0.19 ± 0.04	<0.001^2^
Tyrosine	0.55 ± 0.08	0.80 ± 0.18	<0.001^2^
Valine	1.10 ± 0.15	0.88 ± 0.20	0.004^2^
Methionine	0.55 ± 0.08	0.23 ± 0.05	<0.001^2^
Lysine	2.24 ± 0.31	1.71 ± 0.39	<0.001^2^
Isoleucine	1.24 ± 0.17	0.75 ± 0.17	<0.001^2^
Leucine	2.24 ± 0.31	1.67 ± 0.38	<0.001^2^
Phenylalanine	0.69 ± 0.10	1.09 ± 0.25	<0.001^2^
Tryptophan	0.39 ± 0.05	—	—
∑ Determined amino acids	22.47 ± 3.13	20.71 ± 4.75	0.263
∑ Essential amino acids	10.04 ± 1.40	7.63 ± 1.75	<0.001^2^
∑ Nonessential amino acids	12.43 ± 1.73	13.07 ± 3.0	0.496

Significance was set at *P* ≤ 0.05. Values are means ± SD for *n* = 14 in animal-focused whey protein–supplemented diet (AW-D) and *n* = 13 in plant-focused pea protein–supplemented diet (PP-D). Tryptophan was not measured in PPI and removed from AA sum comparisons. Independent-samples *t* test was run to test for significant differences between groups where significance is denoted.

1 Total daily amount of each amino acid provided by supplemental protein powders (sum of 3 × 0.13 g·kg BM^−1^ servings).

2 *P* < 0.01.

The AW-D and PP-D interventions differed in the proportion of food from animal and plant sources, respectively, for the main evening meal (e.g., pasta with meatballs or vegan meatballs for AW-D and PP-D, respectively). Over the 4 daily meals, the target ratio of animal- to plant-based protein was 75:25 for AW-D and 25:75 for PP-D, respectively. Detailed information on AW-D and PP-D is provided in Table 2, and examples of meal plans for both groups are provided in Supplemental Material 1. Ingredients for all meals were individually weighed by a member of the research team, according to individual protein requirements and predicted total energy expenditure. The diets were designed to allow participants to prepare and heat meals at home, without being overly arduous. For AW-D and PP-D, participants were given 2 set meal plans for each meal/snack, which were rotated daily (i.e., both meal plans were consumed on 5 occasions during the 10-d intervention). Meal rotation was implemented to minimize any boredom or dissatisfaction from consuming identical meals too often. Meals and smoothies were all labeled to ensure consumption on the correct days and were given at each RET visit to provide 2 days’ worth of food intake. Adherence was monitored by asking participants to record the time they consumed food and protein smoothies and returned the empty pots at the next laboratory visit.

**TABLE 2:** Comparison of habitual and controlled 10-d intervention dietary intake.

	AW-D		PP-D		*P* value		
	Habitual	Intervention	Habitual	Intervention	Interaction	Time	Group
Energy (kcal)	1981 ± 468	2133 ± 316	1878 ± 675	2244 ± 429	0.405	0.050	0.978
Carbohydrate (g)	198 ± 48	254.6 ± 41.3	214 ± 81	286 ± 65	0.633	<0.001^1^	0.156
Fat (g)	86 ± 27	81.3 ± 15.9	66 ± 42	72 ± 21	0.453	0.954	0.076
Protein (g)	80 ± 31	74.7 ± 9.7	87 ± 56	78 ± 27	0.831	0.413	0.586
Protein (g·kg BM^−1^)	1.14 ± 0.46	1.04 ± 0.05	1.18 ± 0.6	1.05 ± 0.04	0.906	0.290	0.869
Animal protein (%)	56.3 ± 13.5	74.7 ± 2.0^2^	58.1 ± 16.7	24.9 ± 1.6^2^^,^^3^	<0.001^1^	0.018^1^	<0.001^1^
Fiber (g)	26 ± 6	18 ± 9	24 ± 14	26 ± 7	0.065	0.274	0.182
Salt (g)	2.0 ± 0.7	4.5 ± 1.1	1.5 ± 0.9	5.0 ± 1.3	0.075	<0.001^1^	0.956

Habitual dietary intake recorded on weighed food diaries and intervention diet is from the provided diet for 10 d of the intervention. Values are means ± SD for n=14 participants for AW-D and *n* = 13 participants for PP-D. Significance was set at *P* ≤ 0.05. Two-way analysis of variance (ANOVA) was used to test significant differences between groups and time points.

AW-D, animal-focused whey protein–supplemented diet; PP-D: plant-focused pea protein–supplemented diet.

1 Significant main effect from ANOVA (*P* < 0.05).

2 Significant difference between groups within time point when there is an interaction effect (*P* < 0.05).

3 Significant difference between groups at this time point.

### Resistance exercise training

Participants performed supervised unilateral knee extension RET every other day over the course of the 10-d dietary intervention. The leg to be trained during the 5 RET sessions was randomly selected and counterbalanced for leg dominance within groups. The knee extension machine set-up at each visit was identical to that used during familiarization, on day -2. For each RET session, participants reported to the laboratory after having consumed their breakfast and supplemental smoothie at home ≥2 h prior. The time of each RET session was kept similar for each participant, with the final RET session performed ≥18 h before the postintervention muscle biopsy. For RET sessions, participants completed 10–12 repetitions at ∼75% of their predetermined 1RM for 8 sets on each visit. A member of the research team supervised RET sessions to encourage full range of motion in knee extension/flexion and controlled eccentric and concentric movements. Participants completed an average of 415 ± 20 knee extensions over the 10-d intervention. Following the completion of each set, participants were asked to report a rating of perceived exertion (RPE) using the CR-10 Borg scale [47] with 1 indicating “no exertion” and 10 indicating “maximal exertion.” Additional information on pain and comfort with each RET set was monitored to minimize injury and to ensure that an appropriate muscle anabolic stimulus was delivered. Participants were given 2-min rest between each set to allow for ATP resynthesis and minimize acute postexercise anabolic blunting that may be apparent with very short rest periods [48].

### Analysis

Excluding dietary intake and physical activity data, body composition, and biological sample analysis were performed with the researchers blinded to group and participant IDs. Where appropriate, biological samples from each anonymized group code were analyzed concurrently to minimize any error or bias.

#### Habitual diet

All habitual dietary intakes were recorded in weighted food diaries by participants and analyzed using Nutritics (version 6.0). Food diary entries were spot-checked by an independent researcher for accuracy. Data for all macronutrients and micronutrients were exported into Microsoft Excel (version 16.8; Microsoft), and protein contribution from animal or plant sources was manually delineated by a member of the research team by classifying each food according to whether the constituent elements were primarily animal or plant.

#### Physical activity monitoring

Data collected over the habitual and intervention period were extracted using ActivInsights software and exported into a macro file (version 16.8; Microsoft) to determine physical activity levels during the habitual pre-intervention phase and the 10-d intervention phase.

#### Resting metabolic rate

All data for RER and resting metabolic rate (RMR) were smoothed to 5-s increments and exported as XLS files. Any data deviating by the mean by >2 SDs were excluded from analysis.

#### DXA scanning

DXA outputs for whole*-*body and lower-limb segmental analysis were manually exported into Microsoft Excel (version 16.8; Microsoft).

#### Saliva enrichment

Deuterium (^2^H) enrichment in the body water pool was determined by analysis of daily saliva samples on a Delta V Advantage Isotope Ratio Mass Spectrometer (Thermo Fisher). Saliva samples from days −2, 0, 2, 4, 6, 8, and 10 of the intervention were prepared for ^2^H enrichment analysis. Baseline samples (day −2), taken before D_2_O loading, were prepared neat, without any dilution. The remaining samples, containing D_2_O, were diluted 70× in miliQ water to account for the 70% D_2_O tracer (Cambridge Isotope Laboratories). Standards ranging from 147.5 to 304.0 ppm of ^2^H were used on each run in addition to blanks to find the standard curve. Samples were run in duplicate for each time point after filling each tube with 98% helium gas. Saliva ^2^H enrichment data are presented as APE and expressed relative to enrichment at day −2, before D_2_O loading.

#### Myofibrillar protein extraction

Snap-frozen skeletal muscle samples were weighed (Mettler Toledo, XS205; DualRange) and kept in Eppendorfs on liquid nitrogen. An isolation buffer (2.29 g sucrose, 0.373 g Tris, and 0.372 g EDTA in 80 mL miliQ water) was prepared. Phosphatase inhibitor cocktail (PhosSTOP; Roche) and protease inhibitor (cOmplete mini; Roche) were added for every 10 mL of isolation buffer to prevent unrepresentative phosphorylation changes. Isolation buffer was added in quantities equating to 10× the weight of each muscle sample. Samples were manually homogenized in the detergent-free isolation buffer to minimize destruction to the mitochondrial membrane, preventing contamination of other fractions. Samples were kept on ice, transferred to a 4°C cooled centrifuge (Universal 320R; Hettich), and spun at 700 *g* for 10 min. The resulting supernatant comprised sarcoplasmic proteins was aliquoted into separate Eppendorfs for later immunoblot analysis (described later). To separate the insoluble (extracellular matrix components) and soluble proteins (myofibrillar) from the resulting supernatant, the pellet was washed to remove inhibitors and 1 mL 0.3 M NaOH was added and heated to 50°C to solubilize myofibrillar proteins. Samples were centrifuged at 11,000 *g* for 5 min at 4°C, and the resulting soluble myofibrillar supernatant was transferred to glass tubes. Another 1 mL of NaOH was added to the pellet and centrifuged at 1,000 *g* at 4°C to suspend any residual soluble proteins (i.e., myofibrillar), which were added to the same glass tube. From each glass tube, 50 μL of sample was removed and stored for protein assay to determine the protein content of each myofibrillar fraction before amino acid derivatization. To denature the myofibrillar proteins, 1 mL of 1 M perchloric acid (VWR Avantor) was added to each glass tube to form a pellet. The pellet was cleaned twice with 1 mL of 70% ethanol and centrifuged (Rotanta 460R; Hettich) at 1,240 *g* at 20°C for 5 min with the supernatant discarded each time. To each tube, 2 mL of 6 M hydrochloric acid was then added. Each glass tube was closed with a screw top lid under a nitrogen gas flow to minimize oxidation of samples and vortexed before being placed on a heating block for 16 h at 110°C. Samples were then dried by heating with open lids at 110°C (QBD4; Grant) under a constant nitrogen stream to leave the resulting amino acids.

#### Myofibrillar-bound alanine determination

Derivatization of amino acids and ^2^H-bound alanine were carried out at Maastricht University, Stable Isotope Research Centre, The Netherlands. Prior to insertion on the mass spectrometer, samples were purified by placing columns filled with a negatively charged ion exchange resin. Subsequently, 2 M ammonia was eluted through the column to invert the ionic state of the resin and allow purified amino acids to be collected in glass tubes. Following purification, samples were prepared for insertion on the gas chromatographer (Trace 1310; Thermo Fisher) to separate out amino acids and fed into the isotope ratio mass spectrometer with pyrolysis oven (IR-P-MS, 253 plus; Thermo Fisher). A known standard of the amino acid of interest, alanine, was inserted into the column and ionized creating a reference peak. The ratio of H_2_ and ^2^H at the known spectra peak was evaluated for each myofibrillar sample to determine the amount of ^2^H-bound alanine in the trained and untrained legs, preintervention and postintervention.

#### Myofibrillar FSR

The FSR of myofibrillar protein was determined using the standard precursor–product method, as we have described previously [38]. Total body water (saliva) ^2^H enrichment was used as a surrogate for plasma alanine labeling (precursor) with a correction factor of 3.7 applied as an approximation of the number of hydrogens on alanine, which have been substituted as ^2^H [39]. The within-leg change in myofibrillar-bound ^2^H enrichment of alanine between day 0 and 10 of the intervention was used to calculate myofibrillar FSR using the following equation:

$$\text{FSR}\;\left(\%\;\text{day}\right)=\left(\frac{\text{Em}2-\text{Em}1}{E\text{precursor}\times \Delta t}\right)x\;100$$

where *E*_m1_ and *E*_m2_ are the muscle protein–bound deuterated alanine enrichments on days 0 and 10, respectively; *E*_precursor_ is mean body water enrichment with 3.7 multiplication factor over the incorporation period (day **−**2 to day 9); and *t* represents the time between muscle biopsy sampling, which was first determined in hours to account for subtle differences in the ^2^H incorporation period. FSR in hours was multiplied by 24 to determine daily iMyoPS FSR (%⋅d^−1^).

#### Immunoblotting

Western blot analysis was performed on the sarcoplasmic fraction obtained during myofibrillar protein extraction (described previously). Samples were boiled and concentrated to 2 μg/μL, in accordance with DC protein assay concentrations, in 4× Laemmli sample buffer equally loaded (15 μL per lane) onto 4%–15% gradient precast gels (Criterion TGX; Bio-Rad). Gels were run at 200 V for 1 h in electrophoresis tanks (Bio-Rad). Gels were transferred onto polyvinylidene difluoride (Amersham Cytiva) membranes at 100 V for 1 h. Membranes were blocked in 5% milk and washed in Tris-buffered saline with 0.1% Tween (TBST) before overnight incubation (4°C) with appropriate primary antibodies. The following primary antibodies were used: total mechanistic target of rapamycin [mTOR; CST2983, 1:1000 dilution in 5% bovine serum albumin (BSA) TBST], phosphorylated (p)-mTOR^Ser2448^ (CST2971, 1:1000 dilution in 5% BSA TBST), total protein kinase B (Akt; CST9272, 1:1000 dilution in 5% BSA TBST), p-Akt^Ser473^ (CST4060, 1:1000 dilution in 5% BSA TBST), total eukaryotic elongation factor 2 (eEF2; CST2332, 1:1000 dilution in 5% BSA TBST), and p-eEF2^Thr56^ (CST2331, 1:1000 dilution in 5% BSA TBST); all purchased from Cell Signaling Technology. The following horseradish peroxidase–linked antirabbit (CST7074) IgG dilutions were used; 1:5000 dilution in 3% BSA TBST dilution for total and p-mTOR^Ser2448^ and 1:10,000 in TBST dilution for total Akt, p-Akt^Ser473^, total eEF2, and p-eEF2^Thr56^. Imaging was undertaken using a G:Box Chemi-XR5 (Syngene), and bands were quantified using Fiji ImageJ. Values were corrected to a gel control in the first instance before being corrected to the loading control (Ponceau S). The phosphorylation of each target protein, as a proxy of their activation, was expressed relative to its corresponding total protein signal, with all changes presented relative to the respective preintervention leg.

#### Renal function and lipid profile analysis

Fasted serum samples were collected in SST tubes (BD Vacutainer) on days 0 and 10 of the intervention. Samples were left to clot at room temperature for 10 min before being transferred to ice. Samples were centrifuged at 3500 *g* for 15 min at 4°C, and aliquots were stored at −80°C. Once completed for all participants, samples were analyzed at Heartlands Hospital (University Hospitals Birmingham) on the Alinity c System (Abbott). Urea, creatinine, high-density lipoprotein (HDL) cholesterol, triglycerides, and cholesterol are measured on the Alinity c analyzers by enzymatic assay followed by spectrophotometric measurement. Sodium and potassium were measured by indirect ion-selective electrodes. Samples were run in triplicate, with the average calculated for each analyte.

#### Urinary analysis

Urea and creatinine concentrations were diluted 30-fold and analyzed on the semiautomated Daytona RX+ using the Creatinine and Urea kits (Randox Laboratories). Samples were run in duplicate, and values provided as mmol/L for urea and nmol/L for creatine. To estimate nitrogen loss, derived concentrations of urinary creatinine and urea were expressed relative to their respective molecular weights, converted to g/L, and multiplied by the measured volume of urine excreted. To this, a multiplication factor of 0.37 and 0.46 was applied to creatinine and urea values, respectively, to account for the proportion of the molar mass, which comprised nitrogen. After combining the absolute urea and creatinine values, a further 10% was added to account for nitrogen losses by other means [49]. The amount of protein consumed in the diet was divided by 6.25 as an approximate measure of the nitrogen content of protein. Overall WBNB was determined as the difference between the calculated nitrogen intake and excretion [50] between day 0 and day 10 of the intervention.

### Statistical analysis

Data analysis was performed on Prism GraphPad (V11) and checked for normality using Shapiro–Wilk test on SPSS (version 27; IBM). Between-group differences were evaluated using an independent-samples *t* test. A 2-way repeated-measures analysis of variance (ANOVA) was used to compare within-group (leg; T compared with UT) with a single between-group factor (AW-D compared with PP-D) for iMyoPS and intracellular signaling. For intracellular signaling, post *minus* pre values within legs were used to statistically compare the effects of training and groups, for iMyoPS time is already featured within the calculation of FSR %⋅d^−1^. A 2-way repeated-measures ANOVA was also used to compare dietary intake, renal function markers, blood lipid markers, physical activity, body composition, RER, RMR, leg strength, and architecture over time (days 0 and 10) with a single between-group factor (AW-D and PP-D). Statistical significance was set a priori as *P* ≤ 0.05. Statistically significant main effect or interaction was inspected using Bonferroni post hoc test to correct for multiple comparisons. All values are presented as mean ± SD unless otherwise stated.

## Results

### Anthropometrics and physical activity

Anthropometrics and physical activity characteristics are presented in Table 3. Two-way ANOVA revealed there was no interaction (group × time) for DXA-derived whole-body fat mass, whole-body FFM, or leg-specific FFM (all *P* > 0.05). Similarly, interaction effects were also absent for RER, RMR, and time spent in moderate and vigorous physical activity (MVPA). Irrespective of the intervention, there were main group differences for whole-body FFM, trained and untrained leg FFM, RMR, and MVPA % (all *P* > 0.05).

**TABLE 3:** Participant characteristics at pre- and postintervention.

	AW-D		PP-D		*P* value		
	Preintervention	Postintervention	Preintervention	Postintervention	Interaction	Time	Group
Age (y)	63.9 ± 4.4	—	58.9 ± 7.1^1^	—	—	—	—
Height (m)	1.68 ± 0.1	—	1.70 ± 0.1	—	—	—	—
Body mass (kg)	74.4 ± 17.1	73.4 ± 17.3	73.7 ± 17.8	72.8 ± 18.1	0.991	0.843	0.893
BMI (kg/m^2^)	24.2 ± 2.2	23.9 ± 2.2	24.9 ± 3.6	24.1 ± 4.0	0.767	0.515	0.564
Body fat (%)	31.3 ± 8.3	31.8 ± 8.4	28.7 ± 8.4	29.5 ± 8.8	0.948	0.779	0.293
Whole-body FFM (kg)	44.3 ± 9.5	43.4 ± 9.3	50.7 ± 13.6	50.5 ± 14.1	0.917	0.869	0.048^1^
Trained leg FFM (kg)	6.93 ± 1.66	6.96 ± 1.62	8.47 ± 2.68	8.46 ± 2.80	0.975	0.988	0.020^1^
Untrained leg FFM (kg)	6.99 ± 1.62	6.88 ± 1.60	8.31 ± 2.60	8.27 ± 2.68	0.955	0.903	0.032^1^
RER	0.87 ± 0.06	0.88 ± 0.06	0.87 ± 0.06	0.89 ± 0.07	0.779	0.401	0.779
RMR (kcal)	1446 ± 331	1382 ± 351	1627 ± 579	1596 ± 607	0.330	0.453	0.014^1^
Estimated TEE (kcal)	2161 ± 296	—	2189 ± 465	—	—	—	—
MVPA (%)	17.3 ± 3.8	17.4 ± 4.2	22.0 ± 5.9	20.0 ± 3.5	0.390	0.436	0.004^2^
Nonsedentary time (%)	27.3 ± 15.8	27.6 ± 10.6	31.7 ± 16.4	30.8 ± 10.4	0.873	0.936	0.312

AW-D, animal-focused whey protein–supplemented diet; FFM, fat-free mass; MVPA, moderate and vigorous physical activity; PP-D: plant-focused pea protein–supplemented diet; RER, resting respiratory exchange ratio; RMR, resting metabolic rate; TEE, total energy expenditure.

Significance was set at *P* ≤ 0.05. Values are presented as means ± SD for *n* = 14 participants for AW-D and *n* = 13 participants for PP-D. Two-way repeated-measures analysis of variance was used to evaluate interaction, group and time effects.

1 Significant difference *P* < 0.05.

2 Significant difference *P* < 0.01.

### Dietary intake

Habitual and 10-d intervention dietary intake data are presented in Table 2, the relative amino acid content of each supplement serving is presented in Table 1, and the macronutrient composition of intervention diet meals is presented in Table 4. Dietary protein over the intervention was 1.04 ± 0.05 g·kg BM^−1^·d^−1^ for AW-D and 1.05 ± 0.04 g·kg BM^−1^·d^−1^ for PP-D, with no difference between habitual and intervention protein provision between groups. The provision of WPC and PPI supplementation for AW-D and PP-D, respectively, in addition to discrete differences in whole-food protein sources, ensured that the proportion of animal protein intake was greater for AW-D over PP-D (∼75% vs. ∼25%, respectively; *P* < 0.001). By controlling for the amount and source of protein, the contribution of animal protein (%) significantly differed from habitual values for both groups (*F* = 78.08, *P* < 0.001) and also differed between groups during the intervention (AW-D: +18.4%; PP-D: −33.3%, *P* < 0.001). Irrespective of group, there was a significant main effect for time (difference between habitual and intervention) for carbohydrate (*P* < 0.001) and salt intake (*P* < 0.001) with no overall difference between habitual and intervention diets between groups (interaction all *P* > 0.05) for energy, carbohydrate, fat, protein, or salt, although fibre intake was significantly different between the provided diets (*P* < 0.001). The relative daily total amino acids provided from WPC and PPI (e.g., in AW-D and PP-D, respectively) were comparable (*P* > 0.05), but daily EAA provision was ∼37% higher with WPC than PPI (*P* < 0.001), even when excluding tryptophan, which was not measured/detected in PPI.

**TABLE 4:** Ten-day dietary intervention provision between-group comparison.

	CHO (g)	Fat (g)	Pro (g)	Pro/kg (g)	Fiber (g)	Salt (g)	Energy (Kcal)	AML Pro (%)	PLA Pro (%)
AW-D									
Meal rotation 1	262 ± 40	80.3 ± 14.1	74.6 ± 9.3	1.04 ± 0.05	25.5 ± 2.8	4.10 ± 1.09	2136 ± 302	73.7 ± 3.3	26.3 ± 3.3
Meal rotation 2	248 ± 43	82.3 ± 18.1	74.7 ± 10.3	1.05 ± 0.05	11.7 ± 7.7	4.85 ± 0.89	2131 ± 341	75.1 ± 1.3	24.9 ± 1.3
Average	255 ± 41	81.3 ± 15.9	74.7 ± 9.7	1.04 ± 0.05	18.6 ± 9.0	4.48 ± 1.05	2133 ± 316	74.8 ± 2.7	25.2 ± 2.3
PP-D									
Meal rotation 1	281 ± 98	69.2 ± 29.5	78.0 ± 26.9	1.05 ± 0.28	30.0 ± 10.3	4.67 ± 1.88	2275 ± 718	20.8 ± 4.0	79.2 ± 4.0
Meal rotation 2	292 ± 67	74.5 ± 25.9	78.0 ± 27.1	1.05 ± 0.28	22.1 ± 7.2	5.24 ± 1.78	2213 ± 746	22.1 ± 2.3	77.9 ± 2.3
Average	286 ± 65	71.9 ± 20.6	78.0 ± 17.6	1.05 ± 0.04	26.0 ± 6.8	4.95 ± 1.32	2244 ± 429	21.5 ± 3.3	78.6 ± 3.3
Between groups (*P* value)	0.134	0.157	0.551	0.811	<0.001^1^	0.253	0.443	<0.001^1^	<0.001^1^

AML Pro, animal protein; AW-D, animal-focused whey protein–supplemented diet; CHO, carbohydrates; PLA Pro, plant protein; PP-D, plant-focused pea protein–supplemented diet; ,Pro, protein; Pro/kg, protein per kilogram of body mass.

Meal rotation 1 was provided on days 0, 2, 4, 6, and 8 of the intervention, comprised 4 meals (breakfast, lunch, dinner, and snack, including 3 daily protein–supplemented smoothies). Meal rotation 2 also contains 4 meals with the same 3 daily protein–supplemented smoothies and were provided on days 1, 3, 5, 7, and 9 of the intervention. Significance was set at *P* ≤ 0.05. Data are presented as means ± SD for dietary provision values for *n* = 14 in AW-D and *n* = 13 for PP-D. Independent-samples *t* test was run to compare differences between groups.

1 The average of meal rotations 1 and 2 is significantly different between groups, *P* < 0.01.

### Renal function and serum lipid status

Markers of renal function and lipid status are presented in Table 5. Throughout the intervention, there were no changes in markers of renal function for either group as determined by serum urea, creatinine, sodium, potassium, HDL-cholesterol, triglycerides, LDL-cholesterol, or estimated glomerular filtration rate (eGFR) (interaction all *P* > 0.05). For non-HDL-cholesterol, there was a significant group × time interaction (*F* = 5.69, *P* = 0.027) and time effect (*P* = 0.047), where Bonferroni post hoc tests revealed that non-HDL-cholesterol was significantly lower following the intervention for PP-D only (−12.8% postintervention; *P* = 0.014). The 2-way ANOVA also revealed a main effect of time for total cholesterol (*P* = 0.026), irrespective of group as well as a significant main group difference in eGFR (*P* = 0.049), irrespective of time.

**TABLE 5:** Urinary and serum concentrations of renal and lipid markers before and after the 10-d dietary intervention.

	AW-D		PP-D		*P* value		
	Preintervention	Postintervention	Preintervention	Postintervention	Interaction	Time	Group
Urea (mmol·L^−1^)	5.23 ± 1.55	4.69 ± 0.88	4.96 ± 1.30	4.83 ± 1.07	0.466	0.242	0.887
Creatinine (μmol·L^−1^)	67.3 ± 14.2	66.9 ± 13.3	74.1 ± 15.7	73.7 ± 18.2	0.991	0.673	0.297
Sodium (mmol·L^−1^)	140.2 ± 3.4	139.5 ± 1.5	140.7 ± 5.6	142.4 ± 10.7	0.225	0.610	0.483
Potassium (mmol·L^-−^)	4.87 ± 0.45	4.82 ± 0.32	4.87 ± 0.45	4.92 ± 0.67	0.632	0.967	0.763
HDL-cholesterol (mmol·L^−1^)	1.77 ±0.25	1.69 ± 0.26	1.57 ± 0.46	1.54 ± 0.49	0.455	0.053	0.268
Triglycerides (mmol·L^-−^)	0.82 ± 0.33	0.81 ± 0.35	0.81 ± 0.36	0.69 ± 0.32	0.184	0.093	0.649
Cholesterol (mmol·L^−1^)	5.46 ± 0.95	5.41 ± 0.95	5.47 ± 0.82	4.92 ± 0.78	0.058	0.026^1^	0.500
eGFR (mL·min^−1^/1.73 m^2^)	79.5 ± 4.6	80.6 ± 6.3	87.1 ± 7.5	84.7 ± 9.5	0.073	0.491	0.049^1^
LDL-cholesterol (mmol·L^−1^)	2.87 ± 0.74	2.92 ± 0.73	3.08 ± 0.89	2.69 ± 065	0.077	0.153	0.971
Non-HDL-cholesterol (mmol·L^−1^)	3.69 ± 0.91	3.73 ± 0.84	3.89 ± 0.95	3.38 ± 0.78^2^	0.027^1^	0.047^1^	0.833

ANOVA, analysis of variance; AW-D animal-focused whey protein–supplemented diet; eGFR, estimated glomerular filtration rate; PP-D, plant-focused pea protein–supplemented diet.

Significance was set at *P* ≤ 0.05. Values are means ± SD for *n* = 13 participants for AW-D and *n* = 10 participants for PP-D. Two-way ANOVA was run test for significant differences between time points (pre to post), groups (PP-D vs. AW-D) and interaction (group × time).

1 Significant differences in main ANOVA results (*P* < 0.05).

2 Significant difference within groups between time points when there is an interaction effect (*P* < 0.05).

### Resistance exercise training

Characteristics for RET sessions are presented in Table 6. PP-D had a significantly greater average RPE across all RET sets compared with AW-D (*P* = 0.026). There were no between-group differences in average knee extensor 1RM strength, total training load, volume, and repetition number (Table 6). Following 5 d of unilateral RET, MVC and VA for trained and untrained legs did not differ from preintervention between groups (all interactions *P* > 0.05), with no main effect of time or group for any training parameter (Table 7).

**TABLE 6:** Resistance exercise training characteristics.

	AW-D	PP-D	*P* value
1RM (kg)	45.7 ± 16.6	56.5 ± 30.0	0.232
Target training load (kg)	34.3 ± 12.5	42.3 ± 20.2	0.179
Actual training load (kg)	32.0 ± 10.1	40.4 ± 19.5	0.232
CR-10 RPE (0-10)	6.5 ± 1.1	7.4 ± 1.0	0.026^1^
Total Repetitions (n)	417 ± 22	412 ± 18	0.487
Total Volume (kg)	13355 ± 4182	16735 ±8314	0.203

1RM, 1 repetition maximum; AW-D, animal-focused whey protein–supplemented diet; CR-10 RPE, category ratio 10 scale rating of perceived exertion; PP-D, plant-focused pea protein–supplemented diet.

Significance was set at *P* ≤ 0.05. Values are means ± SD for *n* = 14 participants for AW-D and 13 participants for PP-D during unilateral knee extension resistance exercise training (RET). Total volume was calculated as actual load lifted and repetition number for all sessions. Independent-samples *t* test was run to find any statistical differences in each training variable between groups.

1 Significant difference between PP-D and AW-D (*P* < 0.05).

**TABLE 7:** Skeletal muscle strength, function, and architecture.

	AW-D		PP-D		*P* value		
	Preintervention	Postintervention	Preintervention	Postintervention	Interaction	Time	Group
Trained leg MVC (N)	224.1 ± 62.2	217.4 ± 87.2	262.5 ± 108.6	242.5 ± 127.0	0.521	0.176	0.402
Untrained leg MVC (N)	227.2 ± 81.4	220.4 ± 92.2	257.8 ± 111.1	254.3 ± 137.3	0.919	0.704	0.479
Trained leg VA (%)	79.8 ± 8.1	81.1 ± 8.1	80.9 ± 15.7	79.9 ±14.1	0.237	0.742	0.104
Untrained leg VA (%)	74.5 ± 15.0	71.5 ±13.6	82.6 ± 10.3	84.0 ± 11.0	0.395	0.919	0.992
Trained leg PA (°)	14.9 ± 2.4	13.9 ± 2.4	16.0 ± 3.5	15.5 ± 4.9	0.752	0.283	0.318
Untrained leg PA (°)	14.2 ± 1.4	14.5 ± 2.2	15.1 ± 4.3	16.2 ± 3.4	0.478	0.210	0.281
Trained leg MT (cm)	2.14 ± 0.29	2.10 ± 0.30	2.27 ± 0.38	2.27 ± 0.44	0.690	0.688	0.287
Untrained leg MT (cm)	2.02 ± 0.27	2.08 ± 0.36	2.31 ± 0.34	2.36 ± 0.42	0.746	0.137	0.057

AW-D, animal-focused whey protein–supplemented diet; MT, muscle thickness of m.*Vastus Lateralis*; MVC, maximum voluntary contraction; PA, pennation angle; PP-D, plant-focused pea protein–supplemented diet; VA, voluntary activation.

Significance was set at *P* ≤ 0.05. Values are means ± SD for *n* = 14 participants for AW-D and 13 participants for PP-D for PA and MT. In the trained leg MVC and VA were *n* = 13 for AW-D and 12 for PP-D and the untrained leg *n* = 9 in AW-D and *n* = 12 in PP-D. Two-way analysis of variance was run to test for significant differences between groups and time points.

### ^2^H enrichment and myofibrillar protein synthesis

**FIGURE 3:**
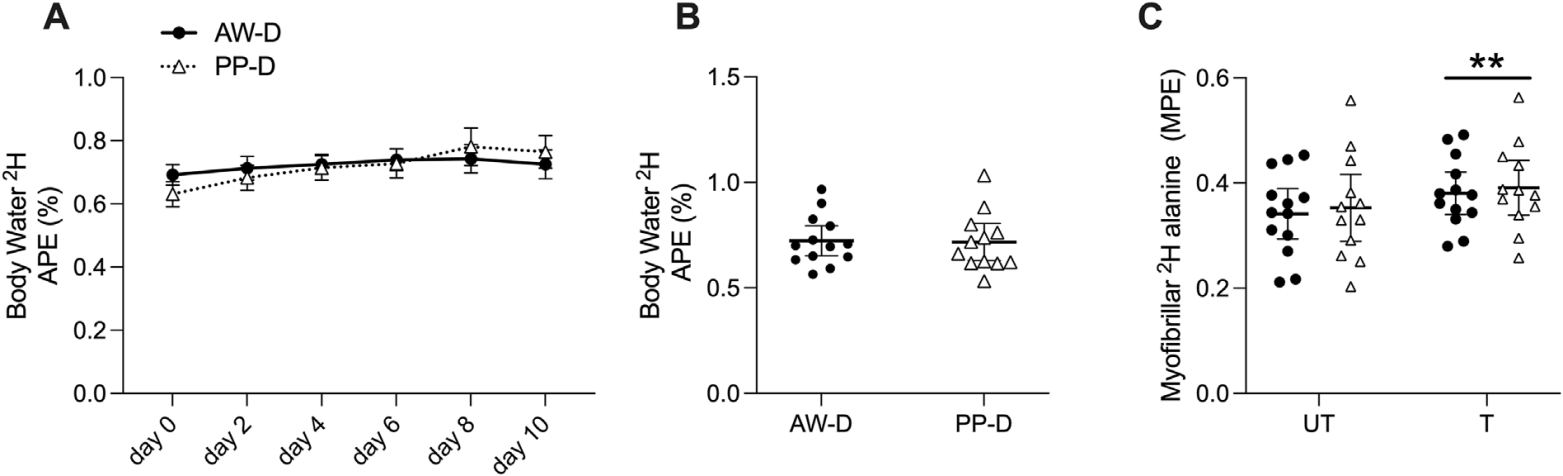
Deuterium enrichment in myofibrillar alanine and body water pool. Time course of saliva enrichment over the 10-d intervention following D_2_O loading in atom percent excess (APE) (A). Two-way analysis of variance showed a significant effect of time (*P* < 0.001) but no main effect for group (*P* = 0.910) or interaction (*P* = 0.214). Mean whole-body water deuterium (^2^H) enrichment from saliva sampling during the intervention (day −1 to day 10 of intervention) following the loading phase (B). An independent-samples *t* test unveiled no significant difference in total body water enrichment between animal-focused whey protein–supplemented diet (AW-D) or plant-focused pea protein–supplemented diet (PP-D) groups (*P* = 0.907). Myofibrillar-bound alanine enrichment in moles percent excess for UT (untrained) and T (trained) leg from days 0 to 10 (C) with no significant interaction (*P* = 0.964) or group effect (*P* = 0.733) but a main effect for training (∗∗*P* < 0.001). Significance was set at *P* ≤ 0.05. Data are shown with individual data points for *n* = 13 for AW-D (black circles) and *n* = 12 for PP-D (white triangles) and plotted as the mean ± SEM.

**FIGURE 4:**
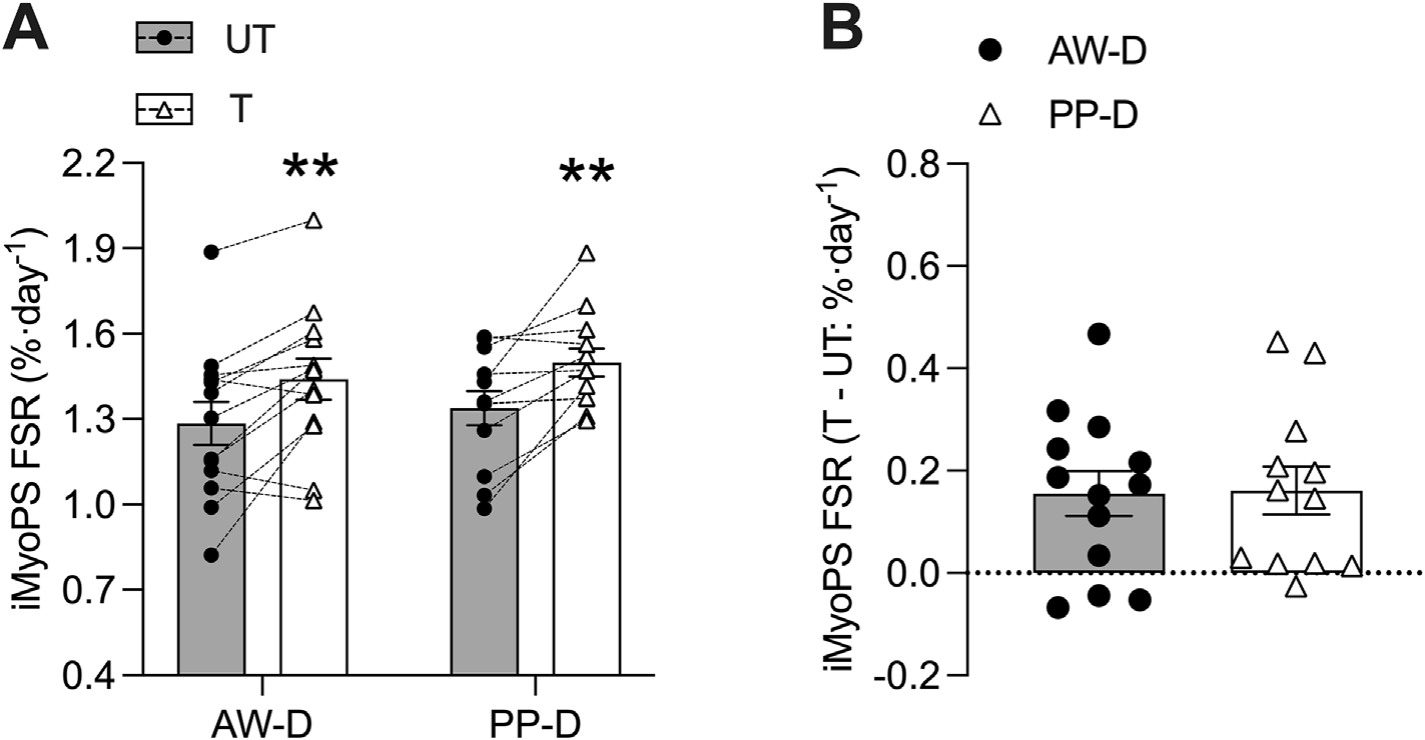
Integrated daily myofibrillar protein synthesis (iMyoPS) rates over 10-d intervention of dietary intervention with divergent protein sources, alone or with combined unilateral resistance exercise training. Daily integrated myofibrillar protein synthesis (iMyoPS) fractional synthesis rate (FSR) over a 10-d period untrained (UT; gray bar and black circles) and trained (T; white bar and white triangles) legs (A) in both animal-focused whey protein–supplemented diet (AW-D) and plant-focused pea protein–supplemented diet (PP-D). Connected black circles and white triangles show individual responses to training, within conditions. Two-way repeated-measures analysis of variance showed no interaction (*P* = 0.936), or main group effect (*P* = 0.527) but a significant effect of training (∗∗*P* < 0.001). Delta change in iMyoPS rates for T relative to UT visualized in (B). An independent-samples *t* test was used to compare differences in iMyoPS between T and UT where there were no differences between groups (*P* > 0.05). Significance was set at *P* ≤ 0.05. Data are shown with individual data points for *n* = 13 for AW-D (circles) and *n* = 12 for PP-D (triangles) and plotted as the mean ± SEM for (A) and (B).

^2^H body water enrichment significantly increased from preintervention to postintervention (day **−**2 to day 10) in AW-D and PP-D (*P* < 0.001 for both) and did not differ between groups (Figure 3A, B). Average body water enrichment over the 10-d intervention did not differ between groups (0.723 ± 0.119 APE and 0.717 ± 0.139 APE for AW-D and PP-D, respectively, *P* = 0.907). Myofibrillar-bound deuterated alanine was significantly increased at day 10 compared with day 0 of the intervention (*P* < 0.001), with no differences between groups or legs (Figure 3C). Myofibrillar deuterated alanine and saliva precursor enrichments enabled the calculation of iMyoPS in trained and untrained legs over the 10-d diet intervention, where there was no group × training interaction for iMyoPS (*P* = 0.956) and no main group effect (*P* = 0.52; Figure 4A). Daily iMyoPS rates were significantly greater (*P* < 0.001) in the trained compared with the untrained leg for AW-D (1.44 ± 0.26 vs. 1.29 ± 0.27 %⋅d^−1^) and PP-D (1.50 ± 0.17 vs. 1.34 ± 0.21 %⋅d^−1^) with no differences between groups, within leg. The absolute difference in the increase in iMyoPS rate between trained and untrained legs was similar between groups (Figure 4B).

### Anabolic signaling phosphorylation

**FIGURE 5:**
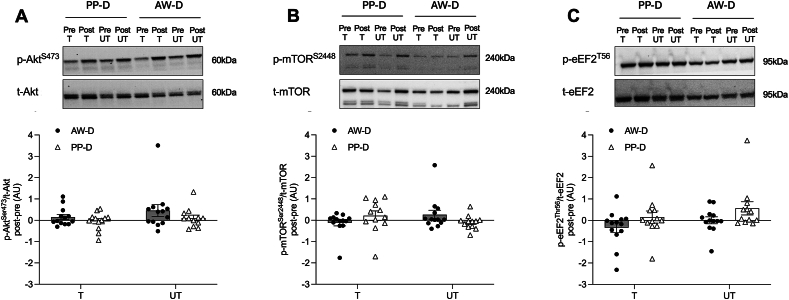
Immunoblots from trained and untrained leg pre- and postintervention for anabolism-related protein targets. Changes in protein expression for phosphorylated (p)-Akt^Ser473^/total (t)-Akt (A), p-mTOR^Ser2448^/t-mTOR (B), and p-eEF2^Thr56^/t-eEF2 (C) in the postabsorptive state expressed as the post- minus preintervention value for trained (T) and untrained (UT) legs. Data are expressed relative to the respective relative total protein, after accounting for loading control and shown as fold change from own leg at pre-, as an internal control, which was normalized to 1. Two-way analysis of variance was used to compare between groups (animal-focused whey-supplemented; AW-D and plant-focused pea supplemented (PP-D) and legs (T and UT). A significant main training effect (P=0.015) for p-eEF2^Thr56^/t-eEF2, which did not remain for the Bonferroni post hoc test (AW-D: P = 0.094, PP-D: P = 0.059). No other significant differences between legs or groups were observed (all P > 0.05). Significance was set at P ≤ 0.05. Data are shown with individual data points and SEM with individual data points for n = 13 for AW-D and n = 12 for PP-D for (A–C). AW-D, animal-focused whey protein–supplemented diet; PP-D, plant-focused pea protein–supplemented diet.

There was a significant training effect for p-eEF2^Thr56^/t-eEF2 (*P* = 0.015), which did not remain after post hoc testing (AW-D: *P* = 0.094; PP-D: *P* = 0.059). There were no other significant main interaction, group, or training effects in phosphorylated over total protein expression for p-Akt^Ser^^473^/t-Akt, p-mTOR^S^^er2448^/t-mTOR, or p-eEF2^Thr56^/t-eEF2 (Figure 5A, C) as determined by immunoblotting (all *P* > 0.05).

### Whole-body nitrogen balance

**FIGURE 6:**
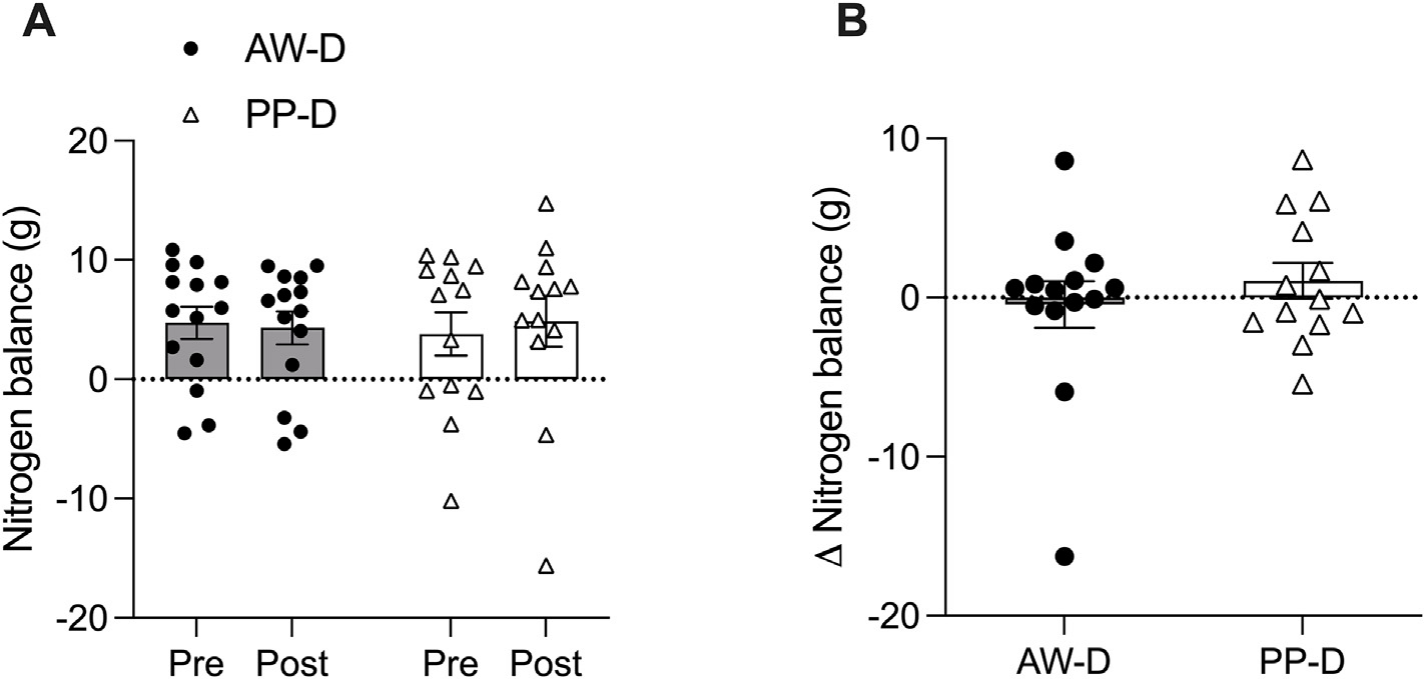
Whole-body nitrogen balance (WBNB) determined from urinary nitrogen excretion and ingested nitrogen (from dietary protein) prior to (pre) and following (post) a 10-d animal-focused whey protein–supplemented diet (AW-D) and plant-focused pea protein–supplemented diet (PP-D) (A). A 2-way ANOVA was used to test for significant differences between AW-D and PP-D, pre- and postintervention with no main interaction (*P* = 0.436), group (*P* = 0.910) or time effect (*P* = 0.746). Delta change in WBNB from pre- to postintervention (B). An independent-samples *t* test was run to test differences between AW-D and PP-D in WBNB change from baseline, with no significant difference between groups (*P* > 0.05). Significance was set at *P* ≤ 0.05. Data are shown with individual data points for *n* = 14 for AW-D (circles) and *n* = 13 for PP-D (triangles) with bars showing the mean ± SEM for (A) and (B). T, trained; UT, untrained.

WBNB was determined as the difference between excreted nitrogen in urea and creatinine from 24 h urine collection over days 0–1 and days 9–10 of the intervention and the level of dietary protein ingestion on these days. There were no significant within- or between-group differences in absolute WBNB (interaction, group, and time effects all *P* > 0.05; Figure 6A) or the relative change in WBNB over the 10-d intervention (*P* > 0.05; Figure 6B).

## Discussion

In the present study, we provided middle-to-older aged adults with a 10-d controlled omnivorous diet containing 1.0 g·kg BM^−1^·d^−1^ protein from either an AW-D or a PP-D, with unilateral leg RET performed every other day. Our novel findings show that daily rates of iMyoPS did not differ in diets containing relatively more animal-derived (AW-D) or more plant-derived (PP-D) protein. These data demonstrate that the primary source of ingested protein in a typical diet of middle-to-older aged adults had no influence on skeletal muscle anabolism. Irrespective of the source of protein in the typical diet of middle-to-older aged adults, RET robustly increased daily iMyoPS with implications for muscle maintenance.

It was hypothesized that the addition of WPC to low-to-moderate protein-containing meals and snacks in AW-D would support greater rates of daily iMyoPS, compared with the addition of PPI to similar meals and snacks in PP-D. However, we did not observe any differences in rested or RET-induced rates of daily iMyoPS between AW-D and PP-D. The absence of any difference in iMyoPS between diets could be due to the lack of any marked difference in postprandial EAA availability in response to the mixed meals and supplements that were provided. Given the relatively high-quality score of PPI compared with other isolated plant-derived proteins [51], postprandial EAA availability may have been sufficient to elicit a comparable muscle anabolic response to WPC in middle-to-older aged adults, particularly when consumed as part of a mixed meal that would address EAA deficiencies [52,53]. Second, PPI and WPC were ingested in smoothies and consumed alongside a whole-food mixed meal or snacks, which would also have altered/slowed EAA digestion and absorption kinetics [54] and potentially influenced acute postprandial muscle anabolism [55]. In this regard, we observed a significantly greater temporal and net postprandial leucine response over 3 h following the AW-D breakfast meal and WPC smoothie, compared with the PP-D breakfast meal and PPI smoothie [30]. It is likely that any divergence in postprandial EAA and leucine availability between AW-D and PP-D meals was not as stark as would be expected if these supplements were consumed in isolation outside of a mixed meal [56]. Nonetheless, although the greater postprandial leucine availability we observed for the AW-D breakfast [30] would likely persist across all lunch and snack events, this did not provide the basis for greater daily iMyoPS rates in the present study. This observation is congruent with McKendry et al. [34] who reported that a high-protein diet with supplemental whey and pea protein provided at breakfast and lunch elicited a similar rate of daily iMyoPS in older males, despite greater postprandial EAA availability in whey-supplemented meals. Similarly, Domić et al. [27] reported a comparable rate of daily mixed MPS with moderate protein-containing whole-food vegan and omnivorous diets.

Habituating to a new (lower) level of dietary protein intake has been reported to alter WBNB, muscle protein turnover, body weight, and RMR [57,58], which could have influenced any potential differences in outcomes between AW-D and PP-D. However, although dietary protein intake was controlled at ∼1g·kg BM^−1^ d^−1^ for the 10-d intervention, and protein intake was theoretically insufficient for maximal postprandial muscle anabolism at breakfast, lunch and snacks [13], participants were consuming similar levels of total daily and per-meal protein in their habitual diet. Based on the absence of any within- or between-group differences in WBNB, RMR, or body mass, we suggest that the controlled diet intervention did not induce any major metabolic perturbations that would mask any potential impact of dietary protein source on the outcomes measured.

Interestingly, PP-D provided ∼44% more fiber than AW-D, largely driven by differences in the whole-food dinner meal, which was devoid of any supplemental protein. Given that plant-derived proteins are rich in fiber [59], this may explain the decrease in non-HDL-cholesterol observed in PP-D [60], which could be interpreted as favorable for long-term cardiometabolic health [61]. However, it is important to highlight that there were no significant interaction effects in blood lipid changes between AW-D and PP-D. Similarly, neither AW-D nor PP-D had any influence on markers of renal function, suggesting that the source of dietary protein in a typical diet of middle-to-older aged adults has no detrimental effects on renal function.

The completion of 5 unilateral RET sessions over the 10-d intervention elicited a greater rate of daily iMyoPS compared with the untrained leg, to a similar extent in AW-D and PP-D. This observation is consistent with data demonstrating that high-volume RET increases the utilization of dietary-derived amino acids for protein synthesis [62] and augments MPS [63] for ≥24 h postexercise [32,64]. Hence, the RET-induced increase in daily iMyoPS observed herein represents the basis for muscle adaptive remodeling over time [65]. Whereas others have failed to detect any increase in iMyoPS over 3 wks of unilateral RET in older adults [6], the greater frequency of RET bouts over a shorter 10-d period in the present study, may explain how we were able to capture a higher rate of daily iMyoPS in the trained leg. Despite greater rates of daily iMyoPS in the trained leg for AW-D and PP-D, no changes in strength, VA, muscle morphology, or architectural properties were detected, likely due to the short time frame of RET [6,[66], [67], [68]]. Similarly, there were no discernible effects of RET on intramuscular anabolic signaling. This is not surprising given that postintervention muscle biopsies were obtained ∼24 h following the final bout of RET to ensure that iMyoPS measures captured the cumulative anabolic response to all RET bouts [64]. Nonetheless, these data emphasize high-volume RET as a potent muscle anabolic stimulus in middle-to-older aged adults consuming a typical protein-containing diet, capable of supporting longer-term muscle adaptive remodeling and offsetting sarcopenia progression [66,69].

In the present study, WPC and PPI supplements provided a moderate dose of protein in a palatable nutrient-rich smoothie as part of a low-to-moderate protein-containing omnivorous meals/snack. This approach, although not representative of the entire whole-food diet of middle-to-older aged adults, allowed us to manipulate the proportion of animal-to-plant protein in omnivorous meals/snack while keeping remaining whole food, energy, and protein intake near identical. Although we cannot rule out that daily iMyoPS may have differed between animal- and plant-focused diets provided exclusively through whole food, others recently reported comparable rates of daily iMyoPS between higher-protein-containing omnivorous and vegan whole-food diets [27]. We posit the present approach of modulating supplemental protein sources within an omnivorous diet holds relevance for scenarios where dietary energy, macronutrient, and protein intake may be compromised (i.e., anorexia of aging, illness, or hospitalization). In this regard, the importance of dietary protein source for muscle anabolism and maintenance in clinical scenarios or in those at an advanced older age warrants further attention. Additionally, the single-blind study design employed in the present study was necessary for the researchers to implement strict control dietary protein source and energy and macronutrient intake. Nonetheless, participants were unable to consistently identify whether they were assigned to AW-D or PP-D. Finally, a crossover study design would have been challenging due to the lengthy time frame of participant involvement (>30 d including washout), prolonged adherence to study control measures, additional muscle biopsies and potential methodological issues arising from extended daily D_2_O consumption (e.g., erroneous precursor pool labeling).

In conclusion, consuming the majority of dietary protein from predominantly animal-derived or plant-derived sources did not modulate rates of daily iMyoPS, WBNB, or intramuscular anabolic signaling in middle-to-older aged adults over a 10-d period. Short-term RET increased daily rates of daily iMyoPS in middle-to-older aged adults, with no influence of the source of protein consumed in the diet. Therefore, in middle-to-older aged adults consuming a typical omnivorous protein-containing diet for 10 d, a higher proportion of plant-derived protein does not compromise muscle anabolism. The importance of dietary protein source (from whole food and supplements) on muscle anabolism and maintenance/accretion with advancing age may be more important in very low protein-containing diets, particularly in clinical scenarios or at a more advanced older age.

## Author contributions

The authors’ responsibilities were as follows – LB, LJCvL, GAW, EIG: conception or design of the work; MK, JIQ, RNM, LMR, AEB, YSE, AL, CA, JMS, JPBG, EIG, GAW, LJCvL, LB: acquisition, analysis, or interpretation of data for the work; MK, LJCvL, GAW, EIG, LB: drafting the work or revising it critically for important intellectual content; MK, LB: agreement to be accountable for all aspects of the work; and all authors: final approval of the version to be published.

## Data availability

Data described in the manuscript, code book, and analytic code will be made available upon request pending approval by the corresponding author.

## Funding

The project was supported by a joint-funded PhD studentship to LB and MK from Volac Whey Nutrition Ltd and the University of Birmingham, United Kingdom.

## Conflict of interest

EIG is a Nutrition Specialist for Volac Whey Nutrition Ltd (now part of Arla Food Ingredients), funders of the research. EIG played a role in the conceptualization of the research and manuscript revisions but no active role in data collection or analysis. LB has received research funding and honoraria for work related to dietary proteins in human health from Dairy UK, Volac International Ltd (Volac Whey Nutrition Ltd), The Hut Group Ltd, Biomega Group, European Whey Processors Association, Danone Nutricia, and the National Dairy Council. LJCvL has received research grants, consulting fees, speaking honoraria, or a combination of these; a full overview on research funding is provided at: https://www.maastrichtuniversity.nl/l.vanloon. The authors have no additional conflicts of interest to declare.
