# Supplementary material for: Resistance training increases myofibrillar protein synthesis in middle-to-older aged adults consuming a typical diet with no influence of protein source: a randomized controlled trial
Source: Am J Clin Nutr. 2025 Apr 25;122(1):122–36. doi: 10.1016/j.ajcnut.2025.04.019 (PMC12308137; doi:10.1016/j.ajcnut.2025.04.019)
Supplement: multimedia component 1 [file mmc1.docx]

## **Supplementary Material 1**

***Example representative diet plan for the 10-day intervention***

For both rotations (consumed on alternating days), semi-skimmed milk and fruit juice were measured to consume throughout each day. Quantities of foods and provided drinks varied according to protein requirements and the energetic needs of each participant. Food sources and quantities at dinner time were changed according to the condition to be primarily plant-derived or animal-derived protein from whole foods only.

***Meal rotation 1***

Breakfast (Whole food and protein smoothie)

Muesli, Greek Yoghurt, Blueberries

Whey or Pea protein-containing smoothie drink

Lunch (Whole food and protein smoothie)

Mixed salad bowl with creaser salad dressing, pretzels, apple or dried tropical fruit pieces

Whey or Pea protein-containing smoothie drink

Dinner (Whole food only)

Jacket potato (heated), mixed vegetables, tuna in sunflower oil, butter.

Greek Yoghurt or Alpro Yoghurt (depending on condition)

Snack (Whole food and protein smoothie)

Rice Cakes, Philadelphia Cheese, fruit pieces, nuts, crisps.

Whey or Pea protein-containing smoothie drink

***Meal rotation 2***

Breakfast (Whole food and protein smoothie)

Porridge oats (made with water or used as part of daily semi-skimmed milk allocation), mango chunks.

Whey or Pea protein-containing smoothie drink

Lunch (Whole food and protein smoothie)

Chicken and Mushroom or Tomato Soup, Wholemeal bread, butter, dried tropical fruit pieces

Whey or Pea protein-containing smoothie drink

Dinner (Whole food only)

Pasta, meatballs (meat-free or beef depending on condition), garlic bread slice, ricotta cheese, sweetcorn and mayonnaise.

Rice pudding, raisins and dried cranberries.

Snack (Whole food and protein smoothie)

Cream crackers, Philadelphia cheese, orange, cranberries and raisins.

Whey or Pea protein-containing smoothie drink
